# Supplementary material for: Protein Nanofibrils from Fava Bean and Its Major Storage Proteins: Formation and Ability to Generate and Stabilise Foams
Source: Foods. 2023 Jan 23;12(3):521. doi: 10.3390/foods12030521 (PMC9914446; doi:10.3390/foods12030521)
Supplement: Supplementary file 1 [file foods-12-00521-s001.zip › foods-2139039-supplementary.pptx]

## Slide 1
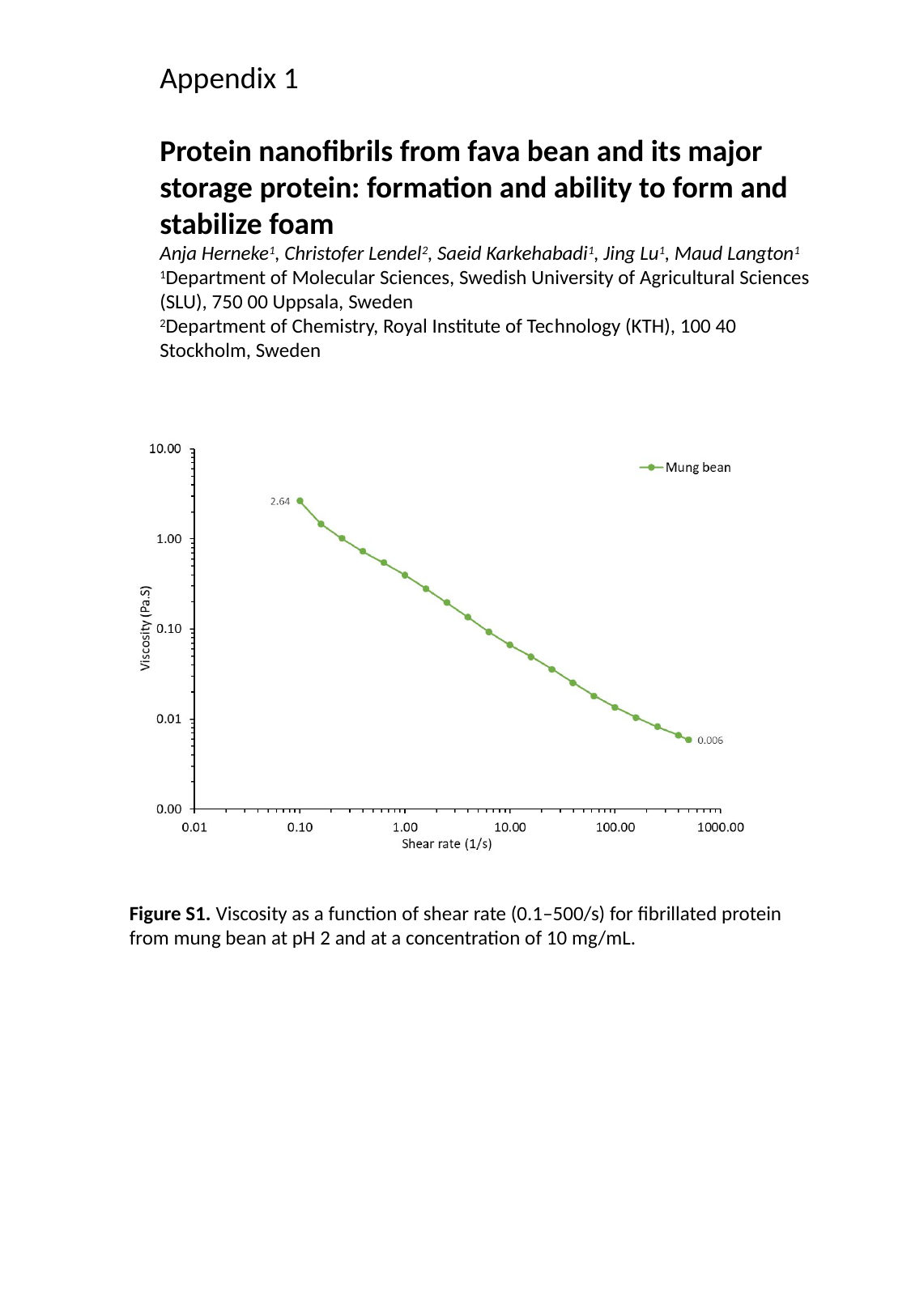

Appendix 1
Protein nanofibrils from fava bean and its major storage protein: formation and ability to form and stabilize foam
Anja Herneke1, Christofer Lendel2, Saeid Karkehabadi1, Jing Lu1, Maud Langton1
1Department of Molecular Sciences, Swedish University of Agricultural Sciences (SLU), 750 00 Uppsala, Sweden
2Department of Chemistry, Royal Institute of Tec­­­­­­hnolog­y (KTH), 100 40 Stockholm, Sweden
Figure S1. Viscosity as a function of shear rate (0.1–500/s) for fibrillated protein from mung bean at pH 2 and at a concentration of 10 mg/mL.

## Slide 2
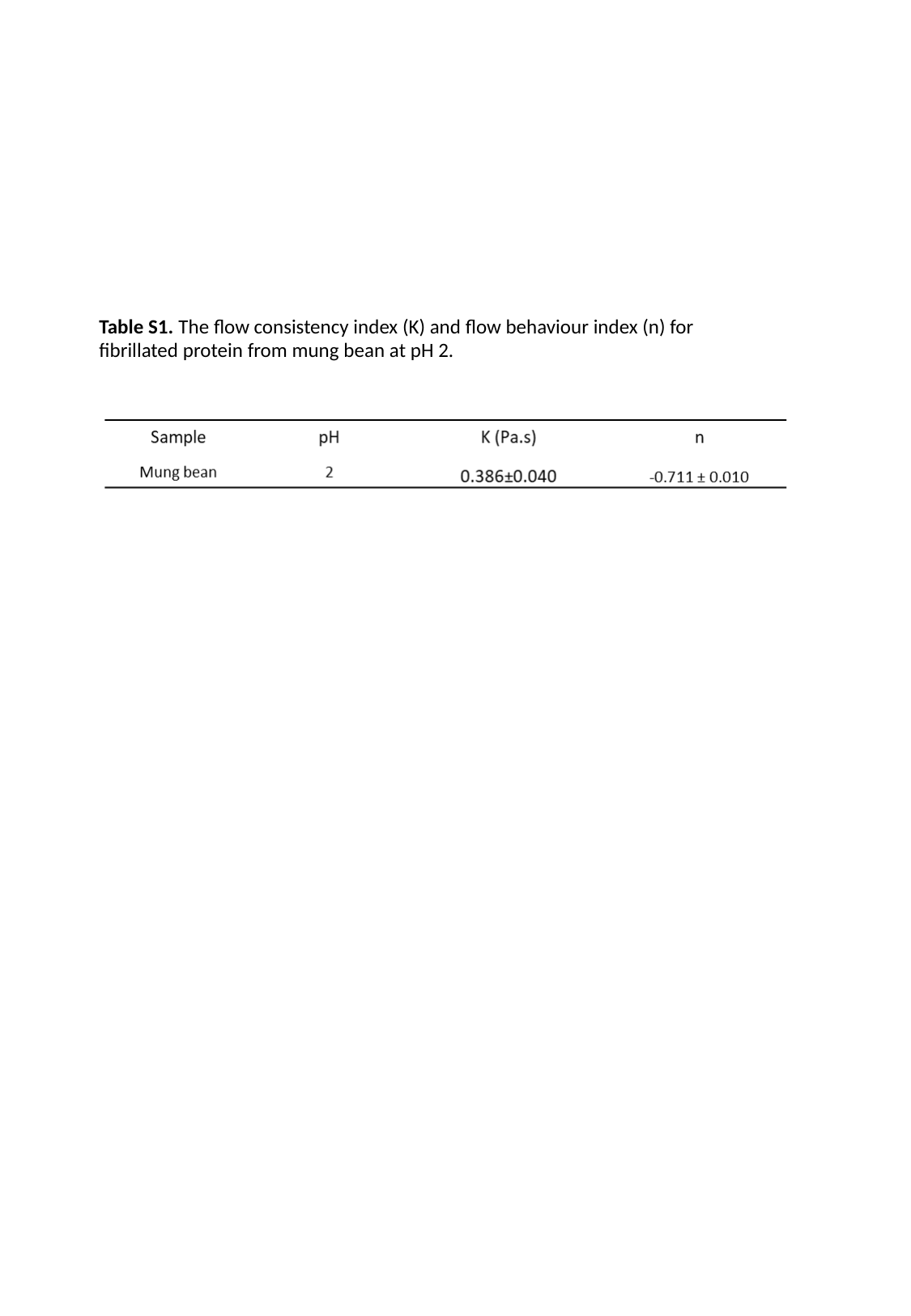

Table S1. The flow consistency index (K) and flow behaviour index (n) for fibrillated protein from mung bean at pH 2.

## Slide 3
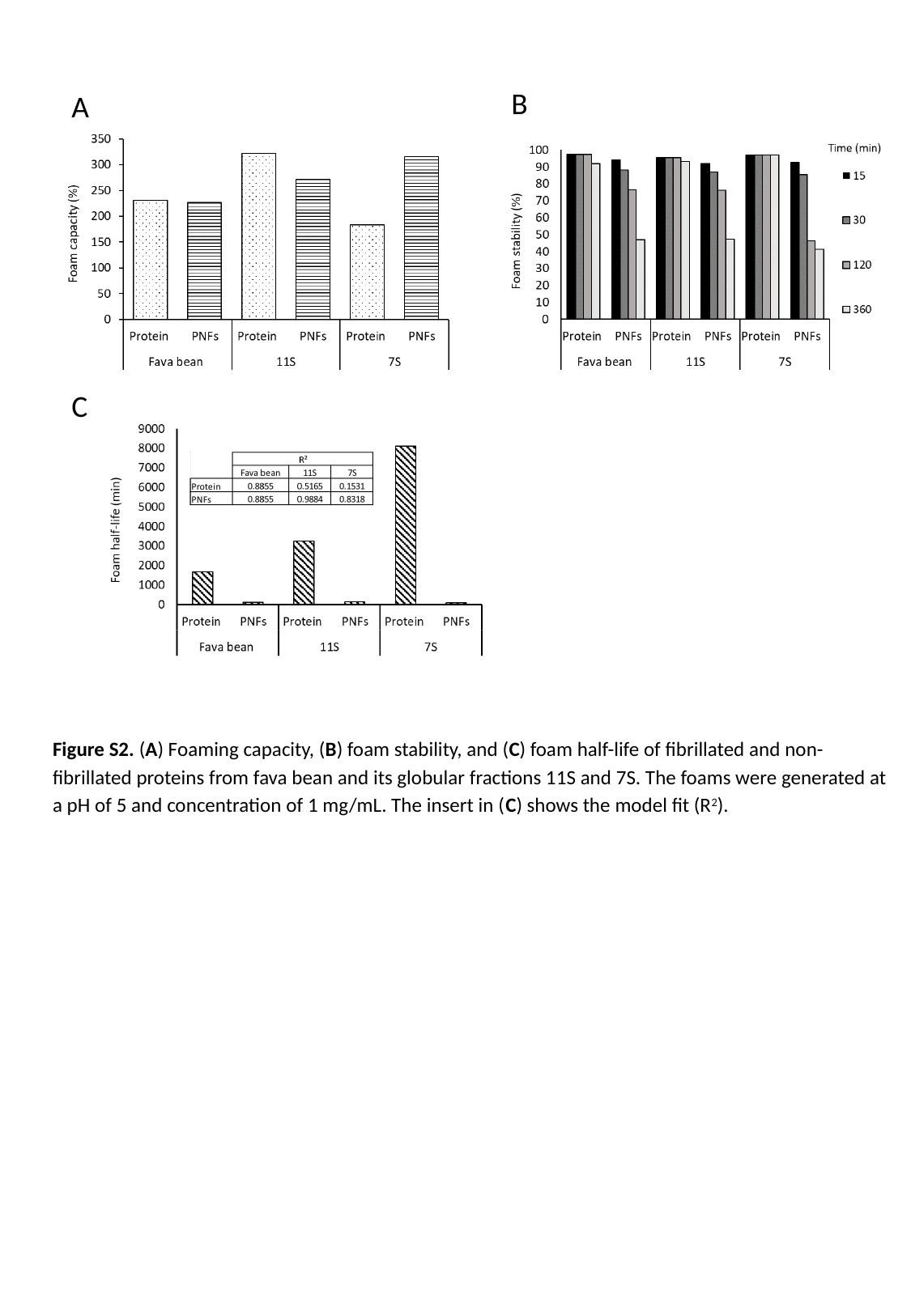

B
A
C
Figure S2. (A) Foaming capacity, (B) foam stability, and (C) foam half-life of fibrillated and non-fibrillated proteins from fava bean and its globular fractions 11S and 7S. The foams were generated at a pH of 5 and concentration of 1 mg/mL. The insert in (C) shows the model fit (R2).
